# Severe Metabolic Acidosis Related to Simultaneous Use of Metformin and Dapagliflozin: A Case Report

**DOI:** 10.7759/cureus.87316

**Published:** 2025-07-05

**Authors:** Irene De la Rosa-Ortega, María Carmen Andreo-López, Alfredo José Pardo-Cabello

**Affiliations:** 1 Internal Medicine, Hospital Universitario San Cecilio, Granada, ESP; 2 Endocrinology, Hospital Universitario San Cecilio, Granada, ESP

**Keywords:** diabetes mellitus in elderly, euglycemic acidosis, isglt-2, metformin induced lactic acidosis, severe diabetic ketoacidosis

## Abstract

We report the case of a 73-year-old woman with type 2 diabetes mellitus (T2D) who developed severe high anion gap metabolic acidosis while on chronic metformin and dapagliflozin therapy. She presented with dehydration following gastrointestinal symptoms, and venous blood gas revealed profound acidosis. Laboratory evaluation showed acute kidney injury and elevated metformin, lactic acid, and ketone blood levels. Despite initial supportive care, she required ICU admission, renal replacement therapy, and vasopressor support. Acidosis resolved within 36 hours. This case highlights the potentially life-threatening risk of combined metformin and sodium-glucose cotransporter 2 (SGLT2) inhibitor use in the setting of volume depletion. Clinicians should monitor renal function and hydration status carefully when prescribing this combination, particularly in elderly patients or those on diuretics.

## Introduction

In recent years we have experienced a revolution in the treatment of type 2 diabetes mellitus (T2D), with the approval of new drugs such as sodium-glucose cotransporter 2 (SGLT2) inhibitors, which are effective in improving glycemic control and offer protection against cardiovascular and renal outcomes [1,2], which is why they are being frequently paired with the first-line therapy for T2D, metformin.

However, both medications have been individually associated with uncommon yet significant acidotic complications that could be life-threatening, including lactic acidosis (with metformin) and euglycemic diabetic ketoacidosis (with SGLT2 inhibitors) [3,4]. Lactic acidosis is defined as a serum lactate concentration above 4 mmol/L and is the most frequent cause of metabolic acidosis in hospitalized patients. Biguanide therapy can be associated with lactic acidosis by various mechanisms (acute overdose, reduced kidney function). Diabetic ketoacidosis (DKA) is one of the most severe acute complications of diabetes, and the most common precipitating factors are infections, omission of insulin therapy, and severe dehydration. It is characterized by metabolic acidosis, increased body ketone concentration, and typically hyperglycemia, but normoglycemic DKA has been described in patients with SGLT2 inhibitors. We present a case of severe acidosis secondary to dehydration in a diabetic patient on chronic dapagliflozin and metformin therapy.

## Case presentation

Our patient was a 73-year-old woman with a relevant prior medical history of T2D, dyslipidemia, and hypertension. Her daily medications included metformin 1000 mg/dapagliflozin 5 mg twice, gliclazide 30 mg, and valsartan 160 mg/hydrochlorothiazide 12.5 mg. Four months earlier, she had normal lab tests, with a serum creatinine of 0.76 mg/dl and an estimated glomerular filtration rate (GFR) of 77 mL/min.

She was admitted to the critical care service due to dyspnea following an episode of vomiting and diarrhea for two days, with obvious signs of dehydration (dry skin and mucous membranes), tachycardia, and tachypnea with accessory muscle use (Kussmaul breathing). Her vital signs on arrival at the emergency room were: heart rate 120 bpm, blood pressure 95/40 mmHg, peripheral oxygen saturation 100%, respiratory rate 28 rpm. Venous blood gas revealed a pH of 6.778, partial pressure of carbon dioxide of 22.5 mmHg, actual bicarbonate of 3.3 mmol/L, anion gap of 41.9 mEq/L and lactic acid of 9.7 mmol/L. Laboratory tests showed she had creatinine of 11.08 mg/dL and urea of ​​262, glucose of 242 mg/dL, glycated hemoglobin (HbA1c) of 8.2%; metformin blood levels were 26921 ng/mL (metformin toxicity is defined as serum levels above 5000 ng/mL) and β-hydroxybutyrate levels were 106 mg/dL (compatible with severe ketosis) (Table 1). Subsequently, anti-IA2 and anti-GAD65 antibodies were analyzed, with negative results, and C-peptide levels were 2.99 ng/mL (reference 0.8-5.2 ng/mL). Limitations include the absence of urinalysis or arterial blood gas.

**Table 1:** Patient’s laboratory test results BUN: blood urea nitrogen; NT-proBNP: N-terminal pro–B-type natriuretic peptide; pCO_2_: partial pressure of carbon dioxide; HCO_3_^-^: bicarbonate; Na: sodium; K: potassium; Cl: chloride

Parameter	Result	Reference range
Venous blood gas		
pH	6.778	7.35 - 7.45
pCO_2_ (mmHg)	22.5	35.0 - 45.0
Actual HCO_3_^-^ (mmol/L)	3.3	22 - 29
Base excess (mmol/L)	-31.3	-2 - 3
Anion gap (mEq/L)	41.9	14 - 18
Lactic acid (mmol/L)	9.7	0.6 - 2.5
Venous blood sample		
Plasma glucose (mg/dL)	242	75 - 115
Hemoglobin A1c (%)	8.2	3 - 6
Metformin (ng/mL)	26921	1000 - 2000 (4 hours after administering)
β-hydroxybutyrate (mg/dL)	106	0 - 4.4
Na (mEq/L)	135	136 - 145
K (mEq/L)	5.2	3.5 – 5.1
Cl (mEq/L)	95	98 - 109
BUN (mg/dL)	122.27	6 - 20
Urea (mg/dL)	262	20 - 43
Creatinine (mg/dL)	11.08	0.5 - 1.1
NT-proBNP (pg/mL)	4373	8 - 300
C-reactive protein (mg/L)	21.2	0.4 - 5
White blood cells (x10^3^/μL)	20.36	3.6 - 10.5
Hemoglobin (g/dL)	14.5	12.5 - 17.2

Markedly elevated anion gap (41.9 mEq/L) in the context of severe acidosis (pH 6.77), high lactate (9.7 mmol/L), and elevated β-hydroxybutyrate (106 mg/dL) confirmed a mixed high anion gap metabolic acidosis, secondary to both metformin-associated lactic acidosis and SGLT2 inhibitors-induced euglycemic ketoacidosis.

**Figure 1:**
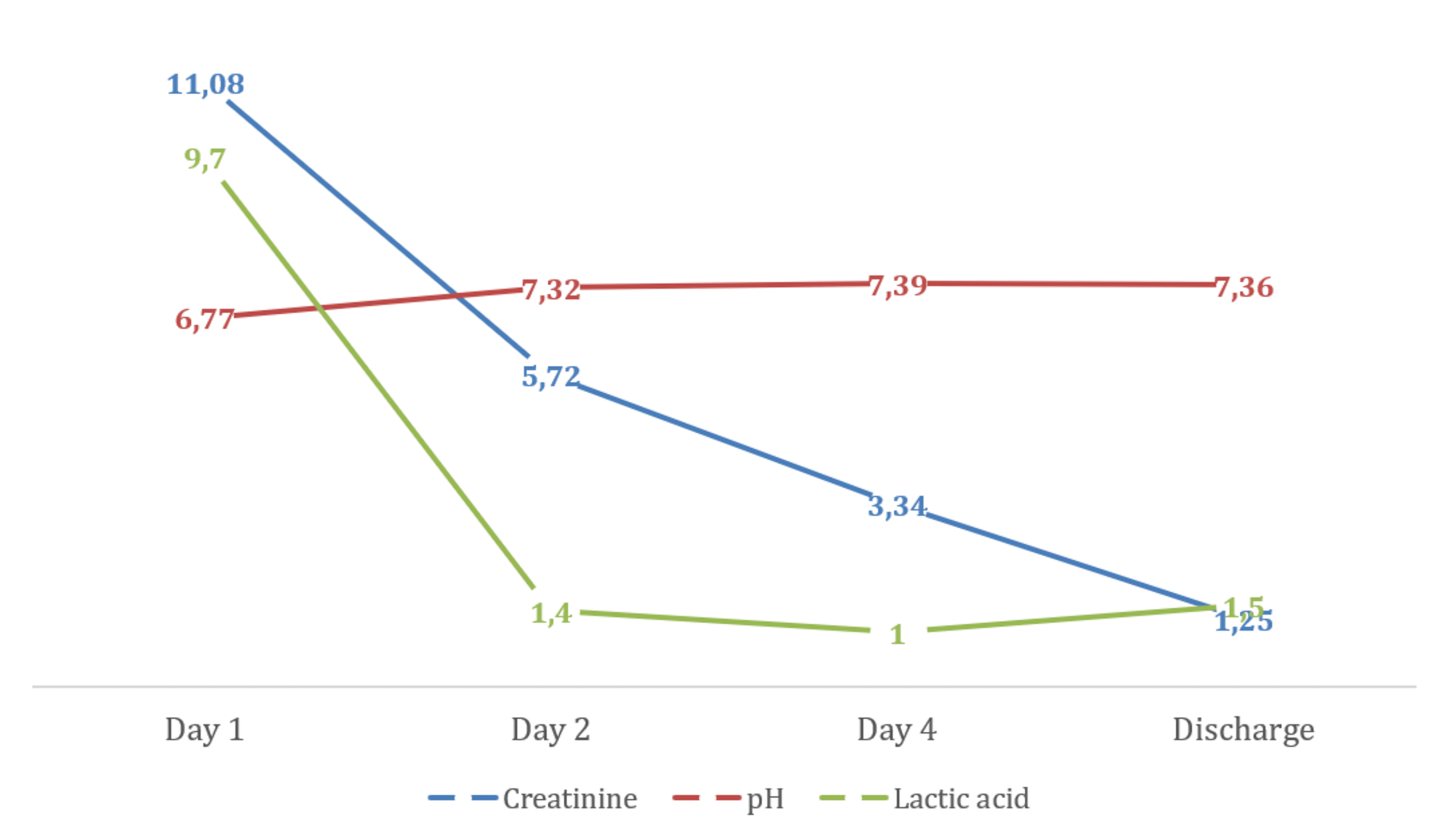
Improvement after renal replacement therapy Creatinine (mg/dL); lactic acid (mmol/L)

She was transferred to the ICU, and they initially administered intravenous sodium bicarbonate and fluids, but due to persisting acidosis and hypotension, continuous renal replacement therapy was initiated and vasoactive agents were required. During her stay in the ICU, our patient’s acidosis progressively improved, and pH normalized after the first 36 hours (Figure 1). The patient was shifted to the medical floor on day three, and by day seven she was discharged on gliclazide and linagliptin, without restarting metformin or dapagliflozin ever since.

## Discussion

Although severe metabolic acidosis in a diabetic patient might suggest diabetic ketoacidosis, mild hyperglycemia, hyperlactacidemia, and acute renal failure would not fully explain our patient’s metabolic status. Furthermore, it is rare for a T2D patient with HbA1c around 8% to develop ketoacidosis, as the pathophysiology of T2D is mainly caused by insulin resistance and not insulinopenia. This leads us to believe our patient’s treatment might be related to lactic acidosis, renal failure, and ketonemia. There are a few similar cases reported in the literature [4-7] in which there is also a mixed cause of severe acidosis, related to both metformin and dapagliflozin. All of these cases were related to dehydration due to fluid restriction, poor oral intake, or increased fluid loss (diarrhea, diuretics), and they all required renal replacement therapy.

SLGT2 inhibitors increase the risk of euglycemic ketoacidosis due to increased hepatic ketogenesis and adipose tissue lipolysis, and they also promote glycosuria, natriuresis, and free water loss, inducing polyuria and increasing the risk of dehydration [4]. They can also worsen metformin-associated lactic acidosis: as volume depletion decreases GFR, dehydration raises the concentration of metformin, which alters hepatic disposal of lactic acid [5]. Moreover, acute kidney injury reduces drug clearance capacity, predisposing to metformin accumulation.

## Conclusions

When combining these two medications, clinicians should be aware of the potential for severe acidosis, especially in cases of serious volume depletions or kidney injuries. If metabolic acidosis does not improve despite intravenous fluids and withdrawal of both drugs, early renal replacement therapy may help prevent further worsening. SGLT2 inhibitors should be introduced gradually in the geriatric population or patients on diuretics who already take metformin. Maintaining proper hydration may help avoid this potentially fatal complication, and temporary discontinuation of SGLT2 inhibitors and/or metformin should be considered in patients with acute gastrointestinal illness, dehydration, or impaired renal function. However, there are few cases similar to ours that have been reported; thus larger registry-based pharmacovigilance datasets are needed to further assess this drug interaction.
